# *Lactobacillus casei* Strain Shirota Enhances the Ability of Geniposide to Activate SIRT1 and Decrease Inflammation and Oxidative Stress in Septic Mice

**DOI:** 10.3389/fphys.2021.678838

**Published:** 2021-09-20

**Authors:** Chao Mai, Li Qiu, Yong Zeng, Xingqin Tan

**Affiliations:** ^1^Department of Emergency, Affiliated Hospital of North Sichuan Medical College, Nanchong, China; ^2^Department of Emergency, The Second Affiliated Hospital of Chongqing Medical University, Chongqing, China; ^3^Department of Anesthesiology Children's Hospital of Chongqing Medical University, National Clinical Research Center for Child Health and Disorders, Ministry of Education Key Laboratory of Child Development and Disorders, Chongqing Key Laboratory of Pediatrics, Chongqing, China

**Keywords:** *Lactobacillus casei*, geniposide, SIRT1, sepsis, mice

## Abstract

*Gardenia jasminoides* Ellis is rich in geniposide, which can be transformed into the anti-oxidant and anti-inflammatory agent genipin. Genipin exhibits greater efficacy than geniposide, but it is unstable and difficult to preserve. In this study, a mouse model for sepsis was established by cecal ligation and puncture, and then we explored the effects and mechanism of *Lactobacillus casei* strain Shirota (LcS) on the enhancement of the ability of geniposide to reduce sepsis and decrease inflammatory and oxidative levels in mice by the regulation of sirtuin type 1 (SIRT1). The mice were evaluated and analyzed by the open field test, Morris water maze test, flow cytometry, kit assay, qPCR, and western blot. The LcS + geniposide increased the survival rate in mice with sepsis, and increased the total travel distance, number of times the mice stood up, amount of time the mice spent grooming their fur, duration in the target quadrant, and crossing area number. The testing of mouse nerve cells showed that LcS + geniposide reduced the rate of nerve cell apoptosis caused by sepsis. LcS + geniposide also decreased the amount of inflammatory-related indicators of TNF-α, IL-6, and IL-1β, and the oxidation-related levels of malondialdehyde (MDA) in the hippocampi of septic mice, and it increased the oxidase activities of superoxide dismutase (SOD) and catalase (CAT). Additionally, LcS + geniposide increased the SOD1, SOD2, and CAT mRNA expression in the hippocampi of mice with sepsis and decreased the expression of TNF-α, IL-1β, NF-κB, and p53 mRNA. LcS+geniposide also increased the SIRT1 protein expression and decreased the Ac-FOXO1, Ac-NF-κB, and Ac-p53 protein expression in the hippocampi of mice with sepsis. We also observed that LcS + geniposide decreased the inflammatory and oxidative damage in the mice with sepsis. The effect of LcS + geniposide was similar to that of the drug dexamethasone and stronger than the effect of geniposide utilized alone. LcS also enhanced the ability of geniposide to activate SIRT1 and decrease the inflammation and oxidative stress in the septic mice, and it achieved an effect same with that obtained by the use of the drug dexamethasone.

## Introduction

Sepsis is an organ dysfunction syndrome caused by an imbalance in response to infection (Jackson, 2004). The main symptoms are chills, fever or low temperature, panic, shortness of breath, and mental state change. The severity of sepsis can increase and develop into septic shock, which can endanger life by leading to organ dysfunction or failure. Sepsis can affect all main organs of the body, and its pathogenesis and developmental mechanism are complex (Rudiger and Singer, 2007). Currently, 30–70% of intensive care unit (ICU) deaths are caused by sepsis and its complications (Zhang et al., 2021), although from 2015 to 2020, the death rate dropped to 30–40% (Raman and Laupland, 2021). Research shows that long-term cognitive impairment remains 1 year after discharge in ~45% of patients with sepsis who were treated and survived, and long-term cognitive impairment continues to affect 10% of patients 3 years after discharge, with inattention and memory loss continuing for up to 6 years after discharge (Wang et al., 2021). Patients with sepsis have impairment of cerebrovascular autoregulation function and dysfunction of cerebral microcirculation. The disease shows the effects of memory loss, cognitive ability and action ability. These symptoms lead to the decline of patients' quality of life and have a negative impact on society and family.

Because of the rapid onset of sepsis, it is difficult to obtain accurate bacterial identification results at the initial stage. Therefore, antibiotics are often used for treatment immediately after discovery, but antibiotics may also produce negative side effects such as antibiotic resistance (Gao M. et al., 2021; Gao X. Q. et al., 2021). Dexamethasone is an essential drug that is commonly used because of its wide range of anti-inflammatory and antiviral effects; therefore, dexamethasone is also used as a basic treatment for sepsis (McNicol et al., 1988; Liu C. X. et al., 2021). In this study, dexamethasone was used as a positive control drug for the treatment of sepsis. Most of the commonly used sepsis drugs result in unwanted side effects. Therefore, the demand for non-toxic and effective drugs or treatment methods with minimal side effects has become an important direction of sepsis research, and correlative studies are highly desirable.

*Gardenia jasminoides* Ellis is a plant in the *Rubiaceae* family and *Gardenia* genus. *Gardenia* fruit is utilized as a traditional Chinese medicine. Modern pharmacological studies have shown that the medicinal effect of the gardenia fruit comes from its organic acids, pigments, and volatile oils, especially the key iridoid glycoside called geniposide, which is an active ingredient (Zhou et al., 2005). Pharmacological studies have shown that genipin is produced when geniposide is hydrolyzed by the β-glucosidase that is produced by intestinal microorganisms (Yang et al., 2011). Genipin exhibits satisfactory anti-oxidant and anti-inflammatory activity, and also regulates lipid metabolism. It exhibits extremely low cytotoxicity, satisfactory biocompatibility and high resistance to degradation (Lin and Yeh, 2012). Although geniposide is relatively easy to extract from gardenia fruits, the hydrolysis of geniposide *via* microorganisms to obtain genipin in industrial production is a low-yield process, and genipin is also difficult to preserve. Therefore, there is little utilization of genipin for disease intervention and treatment (Kim et al., 2017). Studies have shown that during the process of metabolism, some microorganisms can produce β-glucosidase, which hydrolyzes geniposide to produce the anti-cancer substance genipin (Qian et al., 2018).

Considerable research has been performed to study SIRT1, a member of the sirtuin family that is a regulator of a variety of cellular and body processes, namely, metabolism, immune response, and aging, and studies have shown that sepsis is also closely associated with SIRT1 (Li Z. W. et al., 2021). There is a decrease in inflammation, oxidative stress, and even cognitive dysfunction in septic mice *via* SIRT1 activation (Ma et al., 2021). Genipin is unstable and difficult to preserve; therefore, additional research is required to develop a more reasonable application method (Koo et al., 2004). This study also aimed to determine how LcS enhanced the geniposide efficacy in response to sepsis. By establishing animal models and utilizing a variety of experimental methods including molecular biology, this study also elucidated the mechanism of action of LcS + geniposide in ameliorating sepsis *via* SIRT1 activation by observing the changes in inflammation and oxidative stress in mice.

## Materials and Methods

### *In vivo* Experiments With Mice

ICR (Institute of Cancer Research) mice (10 weeks old, male, 120 mice, Kay Biological Technology (Shanghai) Co., Ltd., Shanghai, China) were regularly fed for a week to adapt to the novel environment, and had unlimited access to food and water under temperature-controlled conditions at 21 ± 2°C, humidity at 55 ± 2%, and a 12-h cycle of light/dark. A total of 120 mice were randomly divided into six groups, with 20 mice in each group: (i) normal, (ii) model, (iii) LcS, (iv) geniposide, (v) LcS + geniposide, and (vi) dexamethasone. Except for the normal group, sepsis was established in the remaining groups by cecal ligation and puncture (Gao and Li, 2021). First, the mice with sepsis were anesthetized by intraperitoneal injection of ketamine (15 mg/kg) and xylazine (7.5 mg/kg). Then, the abdomens of the mice were disinfected with topical iodine, and cut longitudinally along the midline by 2 cm so that the cecum could be observed. The intestinal tract was gently pulled out, the feces were slowly squeezed to the cecum, and then the cecum was tied with 5/0 acrylic thread and fixed under a connecting rod in the ileocecum area, while avoiding intestinal obstruction. The cecum was punctured 10 times with a size 18 needle, and the cecum was then squeezed until the fecal overflow became slight. Finally, the cecum was returned to the abdominal cavity, and the wound was closed. An analgesic (butorphanol, 10 mg/kg; Jiangsu Hengrui Pharmaceutical Co., Ltd., Lianyungang, Jiangsu, China) was then administered to the mice. After the completion of the surgical modeling, the mice in each group were treated daily, and normal saline (0.2 ml) was administered by gavage to the mice in the normal and model groups; the LcS group mice were treated with LcS (5 × 10^7^ colony-forming unit (CFU)/kg) by gavage; the geniposide group mice were treated with geniposide (50 mg/kg, purity 99.9%; Shanghai Yuanye Biotechnology Co., Ltd., Shanghai, China) by gavage; the LcS + geniposide group mice were treated with LcS (5 × 10^7^ CFU/kg) and geniposide (50 mg/kg) by gavage; the dexamethasone group mice were treated with dexamethasone (1 mg/kg) by intraperitoneal injection and treated with normal saline (0.2 ml) by gavage; and all the treatments were administered for 1 week. The survival of the mice was observed every day and analyzed after 1 week. A week later, the mice were killed by cervical dislocation, and the tissues and blood of the mice were harvested.

### Open Field Test

Each of the experimental mice was placed alone in a box (length: 100 cm, width: 100 cm, height: 50 cm), and was observed using a video system (Flyde-A; Guangzhou Flyde Biotechnology Co., Ltd., Guangzhou, Guangdong, China) to track over a period of 5 min the total travel distance (m), the number of times the tested mouse stood up, and the number of times it was observed grooming its fur (Tian M. et al., 2019).

### Morris Water Maze Test

The Morris mater maze consists of four visible quadrants in a black circular pool (height: 60 cm, diameter: 150 cm) filled with water (24 ± 2°C). The device has a circular platform (diameter: 10 cm) 2 cm above the water in the center of the third quadrant. The mice entered the pool from four different quadrants in the same order, and the test was performed once a day for 4 days. The mice were observed to see if they could find the platform within 60 s. The experiment continued after each time the mice stayed on the platform for 10 s, and the time it took for the mice to find the platform was recorded. On the fifth day, the platform was removed, and the time the mice spent in the target quadrant and the number of times they crossed the target quadrant were recorded.

### Flow Cytometry

Normal saline at 4°C was added to the mouse hippocampal tissue, which was then prepared as a 1:9 tissue homogenate. The tissue homogenate was filtered with a 200-mesh net, the filter solution was centrifuged (4°C, 3,000 rpm, 5 min), the upper solution was discarded, the cells were resuspended in a Roswell Park Memorial Institute (RPMI) 1640 medium containing 10% calf serum, and the cell concentration was adjusted to 10^6^/ml. Then, 1 ml of the culture medium was centrifuged (4°C, 2,000 rpm, 5 min), the supernatant was discarded, 1 ml of phosphate-buffered saline (PBS) was added, the process was repeated twice, and then the liquid was centrifuged (4°C, 2,000 rpm, 5 min). Next, a 1-ml binding buffer was added to the cells after centrifugation, Annexin V-FITC (5 μl, Invitrogen, Carlsbad, CA, United States) was also added, the solution was incubated in a dark room for 15 min, and then apoptosis was detected by flow cytometry with Accuri C6 (BD Biosciences, San Jose, CA, United States) (Qi et al., 2007).

### Enzyme-Linked Immunosorbent Assay (ELISA)

Normal saline at 4°C was added to the mouse hippocampal tissue, which was then prepared as a 1:9 tissue homogenate. The tissue homogenate was centrifuged (3,000 r/min, 15 min), the supernatant was isolated, and the levels of tumor necrosis factor (TNF)-α (no. H052-1), interleukin (IL)-6 (no. H007-1-1), and IL-1β (no. H002) were measured according to the ELISA kit instructions (Nanjing Jiancheng Bioengineering Institute, Nanjing, China). The assay was completed by a chromogenic reaction of the enzyme (Qian et al., 2018).

### Oxidative Stress Analysis

Normal saline at 4°C was added to the mouse hippocampal tissue, which was then prepared as a 1:9 tissue homogenate. The tissue homogenate was centrifuged (3,000 r/min, 15 min), the supernatant was isolated, and the superoxide dismutase (SOD, no. A001-3-2), catalase (CAT, no. A007-2-1), and malondialdehyde (MDA, no. A003-1-2) levels were measured with WST-1, ultraviolet light, and tetrabutylammonium iodide (TBA) methods according to the kit instructions (Nanjing Jiancheng Bioengineering Institute, Nanjing, China) (Qian et al., 2018).

### Quantitative Polymerase Chain Reaction (qPCR)

Normal saline at 4°C was added to the mouse hippocampal tissue, which was then prepared as a 1:9 tissue homogenate. The RNA was extracted using a TRIzol™ reagent (Invitrogen, Carlsbad, CA, United States), the RNA extract was subsequently diluted to 1 μg/μl, and 1 μl of the diluted RNA extract was then reverse-transcribed using a reverse transcription kit to obtain a cDNA template (Thermo Fisher Scientific, Waltham, MA, United States). The cDNA template (1 μl), SYBR Green PCR Master Mix (10 μl) (Thermo Fisher Scientific, Waltham, MA, United States), sterile distilled water (7 μl), and forward (1 μl) and reverse primers (1 μl) were mixed (Table 1). Then, the mixed solution was reacted (95°C, 60 s), and 40 cycles were performed at 95 (15 s), 55 (30 s), 95 (30 s), and 55°C (35 s) in the PCR instrument (Stepone Plus; Thermo Fisher Scientific, Waltham, MA, United States). After the reaction ended the relative gene expression was calculated *via* the 2^−ΔΔCt^ method using glyceraldehyde-3-phosphate dehydrogenase (GAPDH) as an internal reference (Gao X. Q. et al., 2021).

**Table 1:** Primer sequences of quantitative polymerase chain reaction (qPCR) for this study.

**Gene Name**	**Sequence**
SOD1 (Cu/Zn-SOD)	Forward: 5′-AACCAGTTGTGTTGTCAGGAC-3′
	Reverse: 5′-CCACCATGTTTCTTAGAGTGAGG-3′
SOD2 (Mn-SOD)	Forward: 5′-CAGACCTGCCTTACGACTATGG-3′
	Reverse: 5′-CTCGGTGGCGTTGAGATTGTT-3′
CAT	Forward: 5′-GGAGGCGGGAACCCAATAG-3′
	Reverse: 5′-GTGTGCCATCTCGTCAGTGAA-3′
TNF-α	Forward: 5′-CACGCTCTTCTGTCTACTGAAC-3′
	Reverse: 5′-ATCTGAGTGTGAGGGTCTGG-3′
IL-1β	Forward: 5′-GCAACTGTTCCTGAACTCAACT-3′
	Reverse: 5′-ATCTTTTGGGGTCCGTCAACT-3′
NF-κB	Forward: 5′-CATGTCTCACTCCACAGCT-3′
	Reverse: 5′-CCGGAGAGACCATTGGGA-3′
p53	Forward: 5′-TAACAGTTCCTGCATGGGCGGC-3′
	Reverse: 5′-AGGACAGGCACAAACACGCACC-3′
GAPDH	Forward: 5′-AGGTCGGTGTGAACGGATTTG-3′
	Reverse: 5′-GGGGTCGTTGATGGCAACA-3′

### Western Blot

After collecting the mouse hippocampal tissue and homogenizing it, the cells were lysed with a radioimmunoprecipitation assay (RIPA) lysis solution (Thermo Fisher Scientific, Waltham, MA, United States), the supernatant was removed, and the protein concentration was measured *via* a bicinchoninic acid (BCA) protein detection kit (Thermo Fisher Scientific, Waltham, MA, United States). First, 30 μg of the protein product was separated *via* 12% sodium dodecyl sulfate (SDS) gel electrophoresis (Thermo Fisher Scientific, Waltham, MA, United States) and then transferred to a polyvinylidene fluoride (PVDF) membrane (Thermo Fisher Scientific, Waltham, MA, United States). After it was blocked with 5% bovine serum albumin (BSA) overnight, it was incubated with a rabbit anti-mouse monoclonal antibody on a shaker at 4°C for 2 h. Then, a goat anti-rabbit secondary antibody was added, the membrane was shaken at 4°C for 1 h and then exposed to enhanced chemiluminescence (ECL, Tanon 5200; Shanghai Tanon Technology Co., Ltd., Shanghai, China). Next, the relative expression level of the target protein was analyzed *via* the ImageJ 1.44 software, using β-actin as an internal reference (Luo X. N. et al., 2021).

### High-Performance Liquid Chromatography (HPLC)

After the LcS + geniposide solution (10^7^ CFU/ml LcS and 10 mg/ml) was incubated at 37°C for 12 h, the sample was filtered with a 0.22-μm microporous membrane to obtain a test solution consisting of geniposide and LcS + geniposide. Simultaneously, the sample solution (0.5–1 m) was aspirated into a sample bottle using a disposable needle with a filter membrane. The sample was then tested using the following conditions: Accucore C18 column (1.7 μm, 2.1 mm × 50 mm); 25°C temperature; 35–65% methanol-water for the mobile phase; 0.5 ml/min flow rate; 238 nm detection wavelength; 10 μl sampling volume. According to the chromatogram, the concentration of each substance was calculated from the peak area (Luo H. et al., 2021) (UltiMate 3000 HPLC System; Thermo Fisher Scientific, Waltham, MA, United States).

### Statistical Analysis

Three parallel experiments were conducted for all of the experiments, and the average value was used. The mean values were tabulated as the mean ± standard deviation, and they were analyzed *via* the SPSS v23 statistical software. One-way ANOVA was performed to compare the groups, and *post hoc* comparisons between the groups at the *P* < 0.05 level were observed by Tukey's honestly significant difference test.

## Results

### Survival Status of Mice With Sepsis

**Figure 1:**
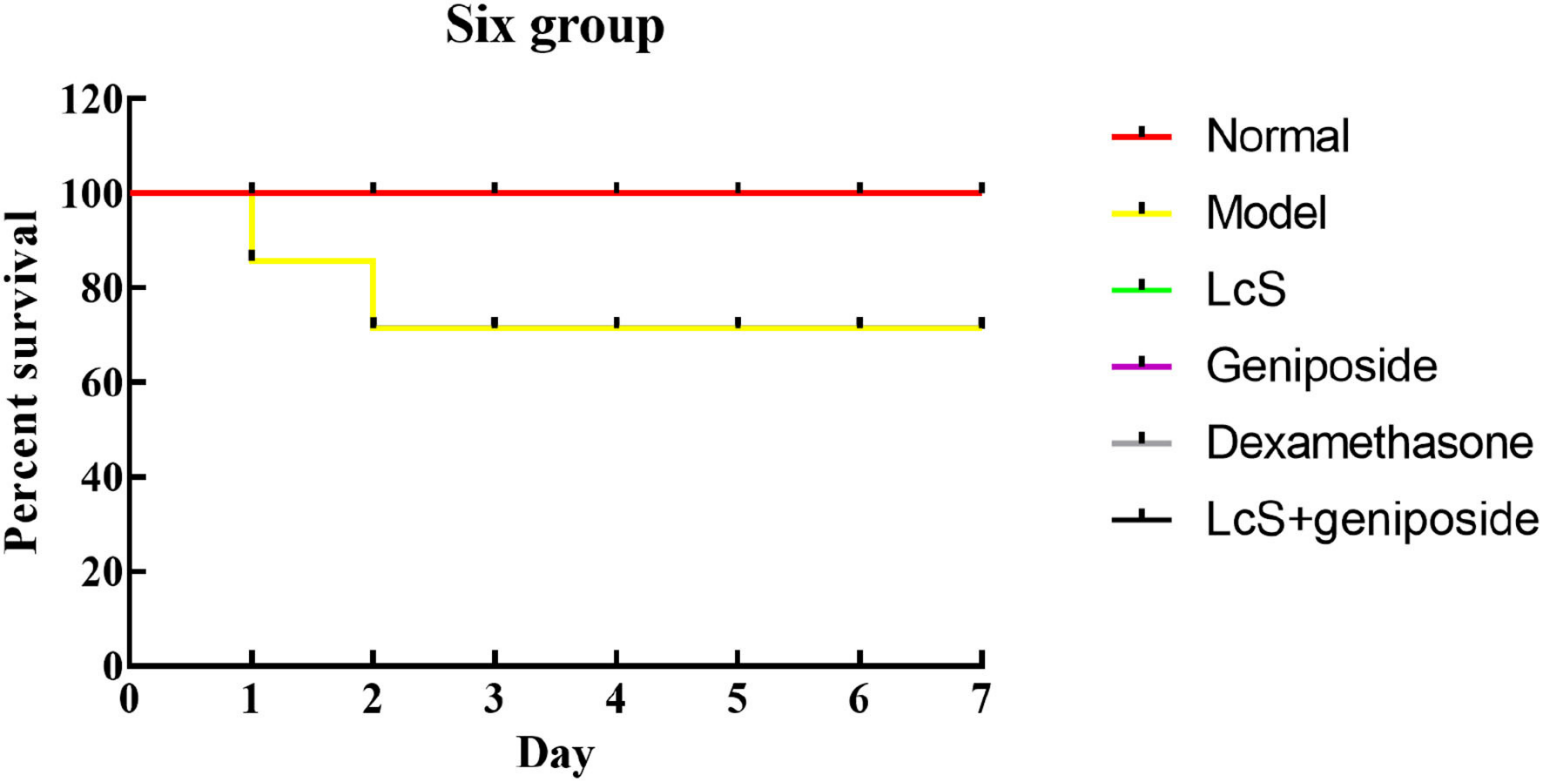
Survival analysis of sepsis mice in Kaplan-Meier curve. Normal: untreated mice; model: mice with induced sepsis; *Lactobacillus casei* strain Shirota (LcS): 5 × 10^7^ colony-forming unit (CFU)/kg-treated mice with sepsis; geniposide: 50 mg/kg geniposide-treated mice with sepsis; dexamethasone: 1 mg/kg dexamethasone-treated mice with sepsis; LcS + geniposide: 5 × 10^7^ CFU/kg LcS and 50 mg/kg geniposide-treated mice with sepsis.

When the experiment began, because of the induction of sepsis, the mice in the other groups died except for those in the normal group, and the greatest number of dead mice originated from the model group (Figure 1, Table 2). Within 48 h, eight mice died in the model group, seven mice died in the LcS group, 6 mice died in the geniposide group, two mice died in the LcS + geniposide group, and three mice in the dexamethasone group died. Greater mouse survival was observed in the mice from the LcS + geniposide and dexamethasone groups as compared with the model group, with similar survival rates in both groups. There was greater survival in the LcS + geniposide and dexamethasone groups as compared with the LcS and geniposide groups. After 48 h, the mice did not continue to die.

**Table 2:** Death rate of mice with sepsis (number of dead mice/total number of mice).

**Group**	**1 day**	**2 day**	**3 day**	**4 day**	**5 day**	**6 day**	**7 day**
Normal	0/20	0/20	0/20	0/20	0/20	0/20	0/20
	4		0	0	0	0	0
Model	3/20	4/20	0/20	0/20	0/20	0/20	0/20
LcS	3/20	3/20	0/20	0/20	0/20	0/20	0/20
Geniposide	1/20	2/20	0/20	0/20	0/20	0/20	0/20
Dexamethasone	1/20	1/20	0/20	0/20	0/20	0/20	0/20
LcS+geniposide	0/20	0/20	0/20	0/20	0/20	0/20	0/20

*Normal: untreated mice; model: mice with induced sepsis; Lactobacillus casei strain Shirota (LcS): 5 × 10^7^ colony-forming unit (CFU)/kg-treated mice with sepsis; geniposide: 50 mg/kg geniposide-treated mice with sepsis; dexamethasone: 1 mg/kg dexamethasone-treated mice with sepsis; LcS + geniposide: 5 × 10^7^ CFU/kg LcS and 50 mg/kg geniposide-treated mice with sepsis*.

### Cognitive Impairment in Mice With Sepsis

**Figure 2:**
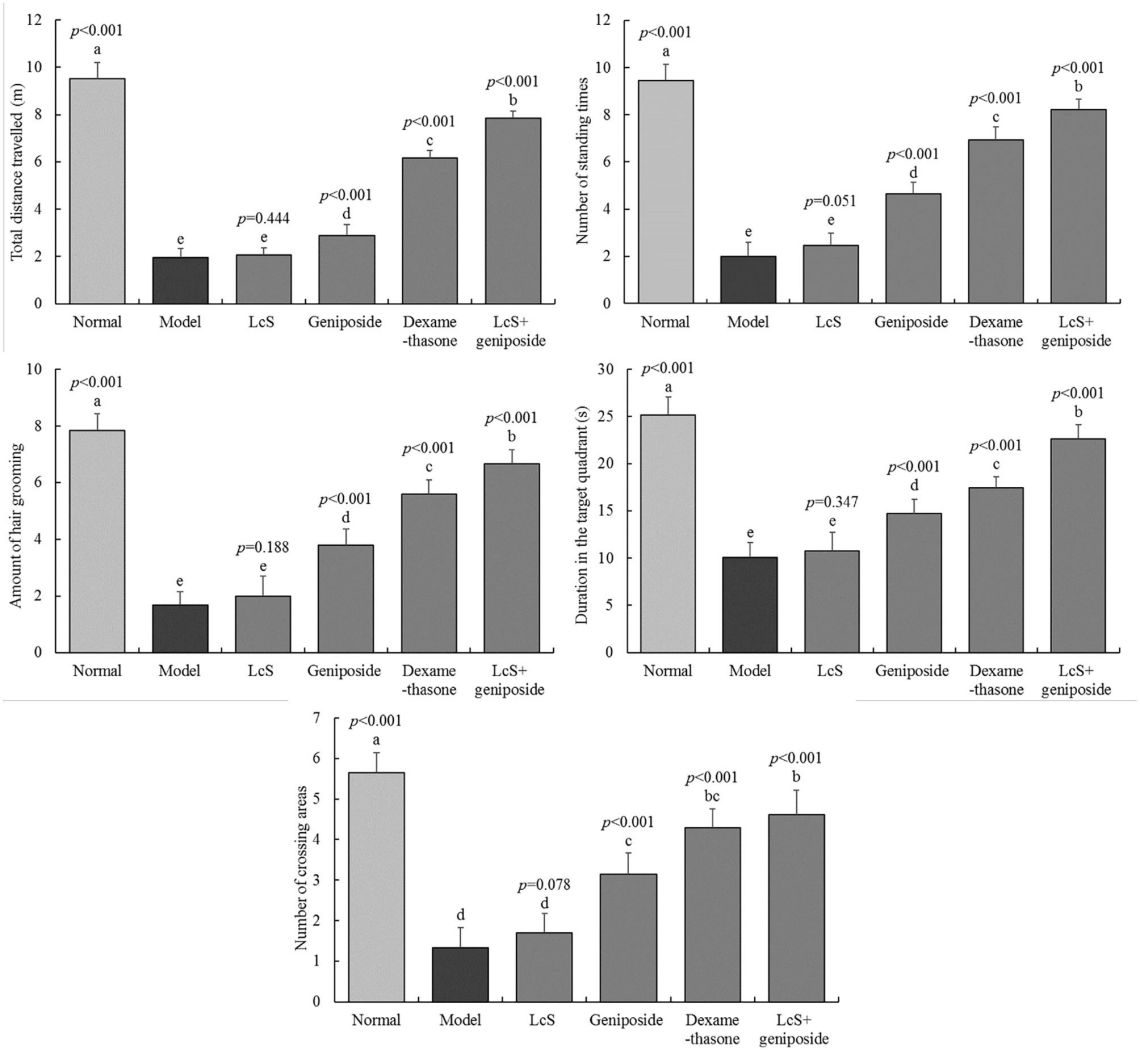
Total distance traveled, number of standing episodes, number of self-grooming episodes, time in the target quadrant, and number of times to cross the area of mice with sepsis in different groups on the seventh day. The mean values are tabulated with mean ± standard deviation, and the *p*-value is the relative situation of the corresponding group to the model group. Normal: untreated mice (*n* = 20), model: mice with induced sepsis (*n* = 12); LcS: 5 × 10^7^ CFU/kg-treated mice with sepsis (*n* = 13); geniposide: 50 mg/kg geniposide-treated mice with sepsis (*n* = 14); dexamethasone: 1 mg/kg dexamethasone-treated mice with sepsis (*n* = 17), LcS + geniposide: 5 × 10^7^ CFU/kg LcS and 50 mg/kg geniposide-treated mice with sepsis (*n* = 18). ^a−e^Mean values with different letters over the bar are significantly different (*P* < 0.05) according to Tukey's honestly significant difference test.

The total travel distance, the number of standing times, the number of times that fur grooming took place, the duration in the target quadrant, and the number of crossing areas for the normal group were 9.53 ± 0.68 m, 9.45 ± 0.69 times, 7.85 ± 0.59 times, 25.15 ± 1.9 s, and 5.65 ±0.49 times, respectively. The indexes for the model group (1.97 ± 0.37 m, 2 ± 0.6 times, 2 ± 0.71 times, 10.08 ± 1.56 s, 1.33 ± 0.49 times, respectively) were significantly reduced, and latency time was significantly increased compared with the normal group (Figure 2). The indexes in the LcS + geniposide (7.86 ± 0.31 m, 8.22 ± 0.43 times, 6.67 ± 0.49 times, 22.61 ± 1.5 s, 4.61 ± 0.61 times, respectively) and dexamethasone (6.15 ± 0.34 m, 6.94 ± 0.56 times, 5.59 ± 0.51 times, 17.47 ± 1.12 s, 4.29 ± 0.47 times, respectively) groups were significantly increased compared with those in the model group, and latency time was decreased. No significant difference was detected between the LcS group and the model group.

### Apoptosis of Mouse Neuronal Cells

**Figure 3:**
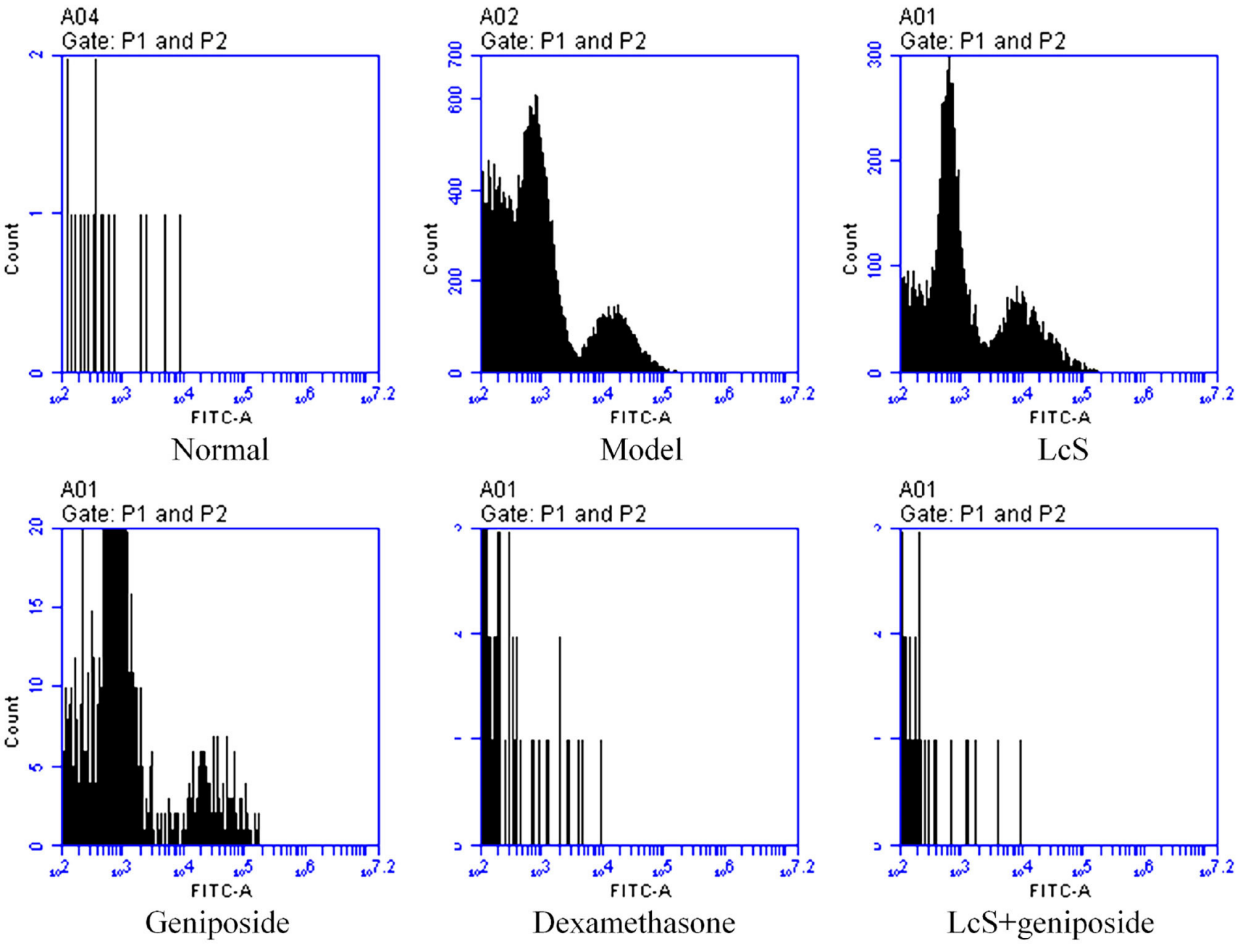
Apoptosis of neuronal cells of septic mice on the seventh day (*n* = 3). Normal: untreated mice; model: mice with induced sepsis; LcS: 5 × 10^7^ CFU/kg-treated mice with sepsis; geniposide: 50 mg/kg geniposide-treated mice with sepsis; dexamethasone: 1 mg/kg dexamethasone-treated mice with sepsis; LcS + geniposide: 5 × 10^7^ CFU/kg LcS and 50 mg/kg geniposide-treated mice with sepsis. ^a−e^Mean values with different letters over the bar are significantly different (*P* < 0.05) according to Tukey's honestly significant difference test.

The mouse neuronal apoptosis rates in the model group were increased compared with the normal group; LcS + geniposide (9.13 ± 0.38%, *p* < 0.001) and dexamethasone (11.53 ± 1.02%, *p* < 0.001) significantly (*p* < 0.05) decreased the neuronal apoptosis rate compared with the model group (38.3 ± 2.72%), and the mouse neuronal apoptosis rates in the LcS + geniposide and dexamethasone groups were lower than those of the geniposide (31.47 ± 0.87%, *p* = 0.014) and LcS (36.7 ± 0.60%, *p* < 0.376) groups. However, no significant difference was detected in the nerve cell apoptosis rate in the LcS group compared with the model group (Figure 3).

### Inflammation in the Hippocampi of Mice

**Figure 4:**
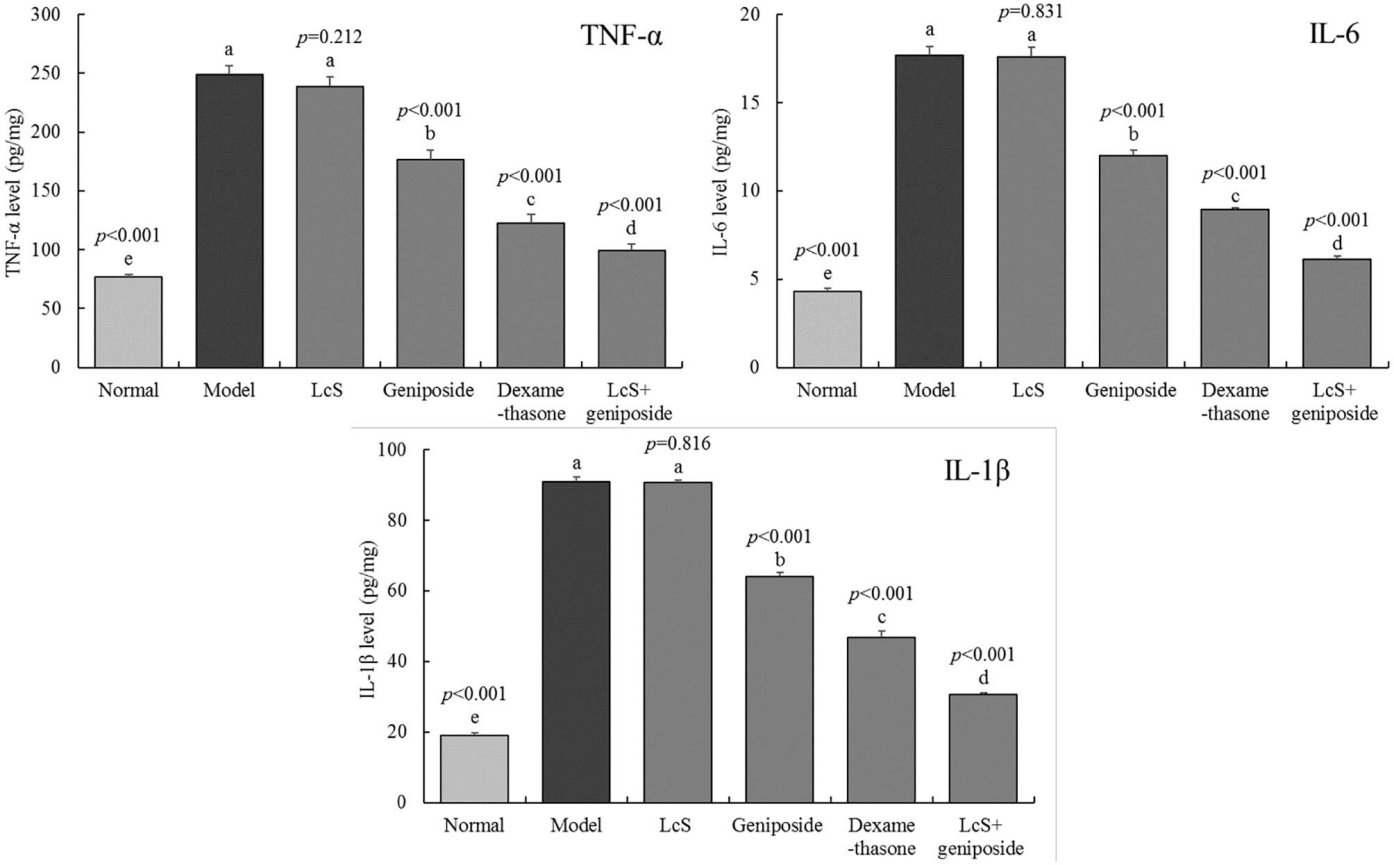
Tumor necrosis factor alpha K(TNF-α), interleukin-6 (IL-6), and interleukin-1 beta (IL-1β) cytokines levels of the hippocampus of mice with sepsis on the seventh day (*n* = 8). The mean values are tabulated with mean ± standard deviation, and the *p*-value is the relative situation of the corresponding group to the model group. Normal: untreated mice; model: mice with induced sepsis; LcS: 5 × 10^7^ CFU/kg-treated mice with sepsis; geniposide: 50 mg/kg geniposide-treated mice with sepsis; dexamethasone: 1 mg/kg dexamethasone-treated mice with sepsis; LcS + geniposide: 5 × 10^7^ CFU/kg LcS and 50 mg/kg geniposide-treated mice with sepsis. ^a−e^Mean values with different letters over the bar are significantly different (*P* < 0.05) according to Tukey's honestly significant difference test.

The model group mice exhibited the highest inflammatory cytokine levels of TNF-α (248.69 ± 7.91 pg/mg tissue), IL-6 (17.7 ± 0.49 pg/mg tissue), and IL-1β (90.89 ± 1.3 pg/mg tissue) (Figure 4), while the normal group exhibited the lowest levels (76.87 ± 1.85, 4.31 ± 0.19, and 19.03 ± 0.6 pg/mg tissue, respectively). Geniposide, LcS + geniposide, and dexamethasone significantly reduced the TNF-α (176.71 ± 8.31, 99.63 ± 5.39, and 122.86 ± 7.25 pg/mg tissue), IL-6 (11.98 ± 0.34, 6.13 ± 0.17 and 8.94 ± 0.1 pg/mg tissue), and IL-1β (64.16 ± 0.97, 30.54 ± 0.61 and 46.85 ± 1.69 pg/mg tissue) levels in the hippocampi of the mice with sepsis. LcS + geniposide and dexamethasone were the most effective, which resulted in these levels being closer to those of the normal group, while the effects of LcS + geniposide and dexamethasone were stronger than those of LcS and geniposide.

### Oxidative Stress Response in the Mouse Hippocampus

**Figure 5:**
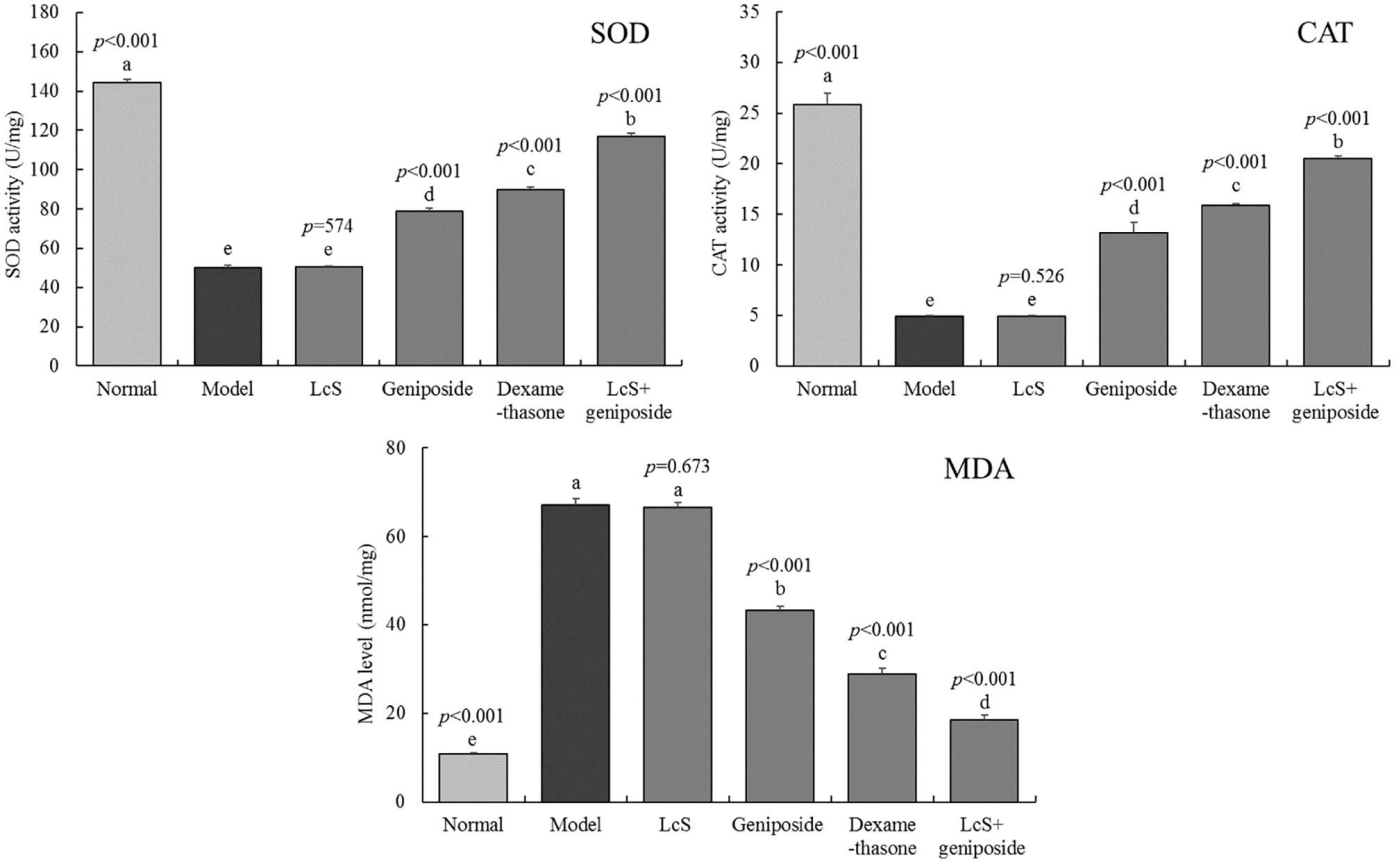
Superoxide dismutase (SOD), catalase (CAT), and malondialdehyde (MDA) levels of the hippocampus of mice with sepsis on the seventh day (*n* = 8). The mean values are tabulated with mean ± standard deviation, and the *p*-value is the relative situation of the corresponding group to the model group. Normal: untreated mice, model: mice with induced sepsis; LcS: 5 × 10^7^ CFU/kg-treated mice with sepsis; geniposide: 50 mg/kg geniposide-treated mice with sepsis; dexamethasone: 1 mg/kg dexamethasone-treated mice with sepsis; LcS + geniposide: 5 × 10^7^ CFU/kg LcS and 50 mg/kg geniposide-treated mice with sepsis. ^a−e^ Mean values with different letters over the bar are significantly different (*P* < 0.05) according to Tukey's honestly significant difference test.

The enzymatic activity (SOD, CAT) in the normal group mouse hippocampi (144.3 ± 1.71 and 25.89 ± 1.1 U/mg) was the strongest (Figure 5). The MDA levels (10.8 ± 0.25 mmol/mg) were the lowest, while the mice in the model group exhibited a reverse trend: the levels of MDA (67.03 ± 1.51 mmol/mg) in the mice were the highest, and the enzymatic activity (SOD, CAT) was the lowest (50.2 ± 1.16 and 4.89 ± 0.11 U/mg). After LcS + geniposide (LcS + geniposide group) and dexamethasone (dexamethasone group) treatment, the enzymatic activity (SOD, 116.9 ± 150 and 89.8 ± 1.1 U/mg; CAT, 20.48 ± 0.25 and 15.86 ± 0.23 U/mg) and MDA levels (18.54 ± 1.09 and 28.91 ± 1.28 mmol/mg) in the hippocampi of mice approached those in the normal group, and the effects were significantly stronger than those of geniposide, with LcS exhibiting the lowest effects.

### mRNA Expression in the Mouse Hippocampus

**Figure 6:**
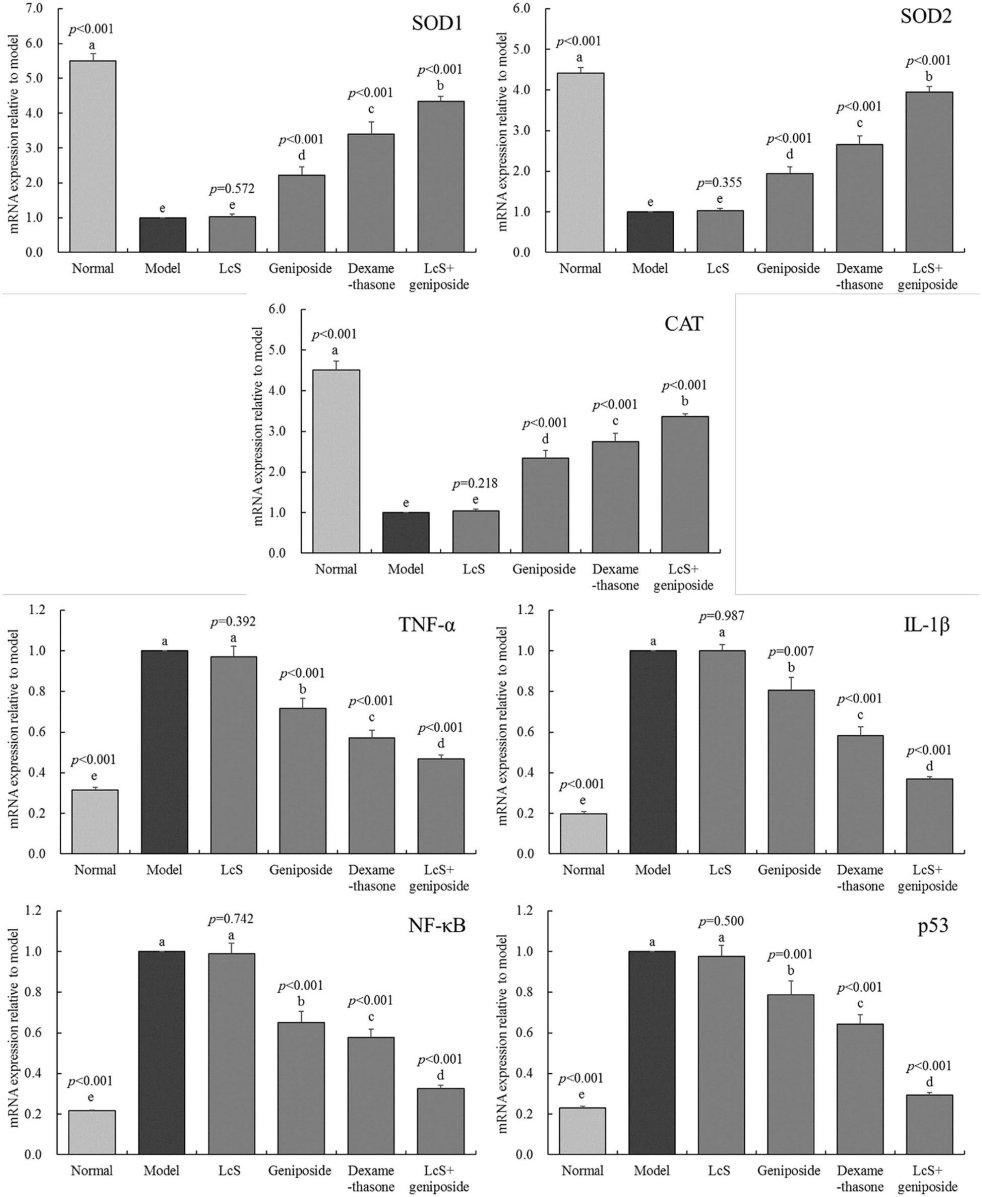
SOD1, SOD2, CAT, MDA, TNF-α, IL-1β, nuclear factor kappa beta (NF-κB and p53 mRNA expression of the hippocampus of mice with sepsis on the seventh day (*n* = 3). The mean values are tabulated with mean ± standard deviation, and the *p*-value is the relative situation of the corresponding group to the model group. Normal: untreated mice; model: mice with induced sepsis, LcS: 5 × 10^7^ CFU/kg-treated mice with sepsis; geniposide: 50 mg/kg geniposide-treated mice with sepsis; dexamethasone: 1 mg/kg dexamethasone-treated mice with sepsis; LcS + geniposide: 5 × 10^7^ CFU/kg LcS and 50 mg/kg geniposide-treated mice with sepsis. ^a−e^ Mean values with different letters over the bar are significantly different (*P* < 0.05) according to Tukey's honestly significant difference test.

The SOD1, SOD2, and CAT mRNA expression in the hippocampi of the model group mice was lower than that of the other groups (Figure 6), while the TNF-α, IL-1β, NF-κB, and p53 mRNA expression was stronger than that measured in the other groups. Geniposide, LcS + geniposide, and dexamethasone increased the mRNA expression of SOD1 (2.23 ± 0.23-, 4.35 ± 0.13-, and 3.39 ± 0.36-fold of the model), SOD2 (1.94 ± 0.17-, 3.94 ± 0.14-, and 2.65 ± 0.22-fold of the model), and CAT (2.34 ± 0.19-, 3.36 ± 0.08-, and 2.75 ± 0.21-fold of the model) in the hippocampi of the mice with sepsis and decreased the TNF-α mRNA expression (0.72 ± 0.05-, 0.47 ± 0.02-, and 0.57 ± 0.04-fold of the model), IL-1β (0.81 ± 0.07-, 0.37 ± 0.01-, and 0.58 ± 0.04-fold of the model), NF-κB (0.65 ± 0.05-, 0.33 ± 0.01-, and 0.58 ± 0.04-fold of the model), and p53 (0.79 ± 0.07-, 0.29 ± 0.01-, and 0.64 ± 0.05-fold of the model) levels, while LcS + geniposide and dexamethasone exhibited the strongest effects as compared with geniposide and LcS.

### Protein Expression in the Hippocampal Tissue of Mice

**Figure 7:**
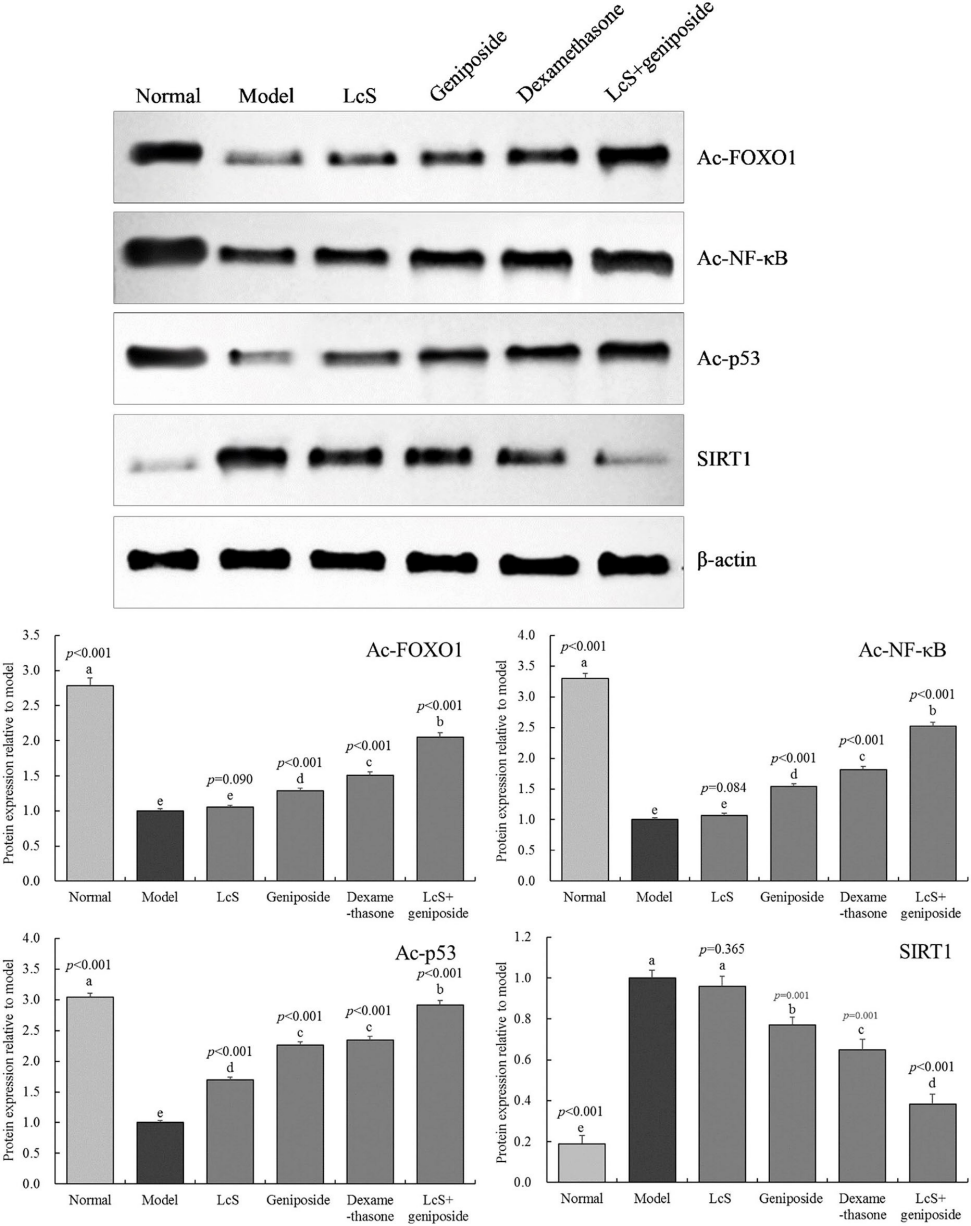
Ac-forkhead box O1 (Ac-FOXO1), Ac- nuclear factor kappa beta (NF-κB), Ac-p53, and sirtuin type 1 (SIRT1) protein expression of the hippocampus of mice with sepsis on the seventh day (*n* = 3). The mean values are tabulated with mean ± standard deviation, and the *p*-value is the relative situation of the corresponding group to the model group. Normal: untreated mice; model: mice with induced sepsis; LcS: 5 × 10^7^ CFU/kg-treated mice with sepsis; geniposide: 50 mg/kg geniposide-treated mice with sepsis; dexamethasone: 1 mg/kg dexamethasone-treated mice with sepsis; LcS + geniposide: 5 × 10^7^ CFU/kg LcS and 50 mg/kg geniposide-treated mice with sepsis. ^a−e^ Mean values with different letters over the bar are significantly different (*P* < 0.05) according to Tukey's honestly significant difference test.

Figure 7 shows that the Ac-FOXO1, Ac-NF-κB, and Ac-p53 protein expression in the model group hippocampal tissue was the weakest, while the protein expression of SIRT1 was the strongest. When compared with the model group, geniposide, LcS + geniposide, and dexamethasone increased the protein expression of SIRT1 (0.77 ± 0.04, 0.38 ± 0.05, and 0.65 ± 0.05-folds of the model group, respectively) and decreased the protein expression of Ac-FOXO1 (1.28 ± 0.04-, 2.05 ± 0.06-, and 1.51 ± 0.05-fold of the model group, respectively), Ac-NF-κB (1.54 ± 0.05-, 2.52 ± 0.06-, and 1.81 ± 0.06-fold of the model group, respectively), and Ac-p53 (2.26 ± 0.06-, 2.92 ± 0.07-, and 2.34 ± 0.07-fold of the model group) in the mouse hippocampus. LcS + geniposide and dexamethasone exerted stronger effects on these proteins as compared with geniposide, while LcS + geniposide and dexamethasone treatment resulted in Ac-FOXO1, Ac-NF-κB, Ac-p53, and SIRT1 protein expression in mice with sepsis that was closest to that of the normal group. The protein expression in the LcS + geniposide and dexamethasone groups was stronger than that in the geniposide or LcS group.

### LcS Conversion Effect of Genipin

**Figure 8:**
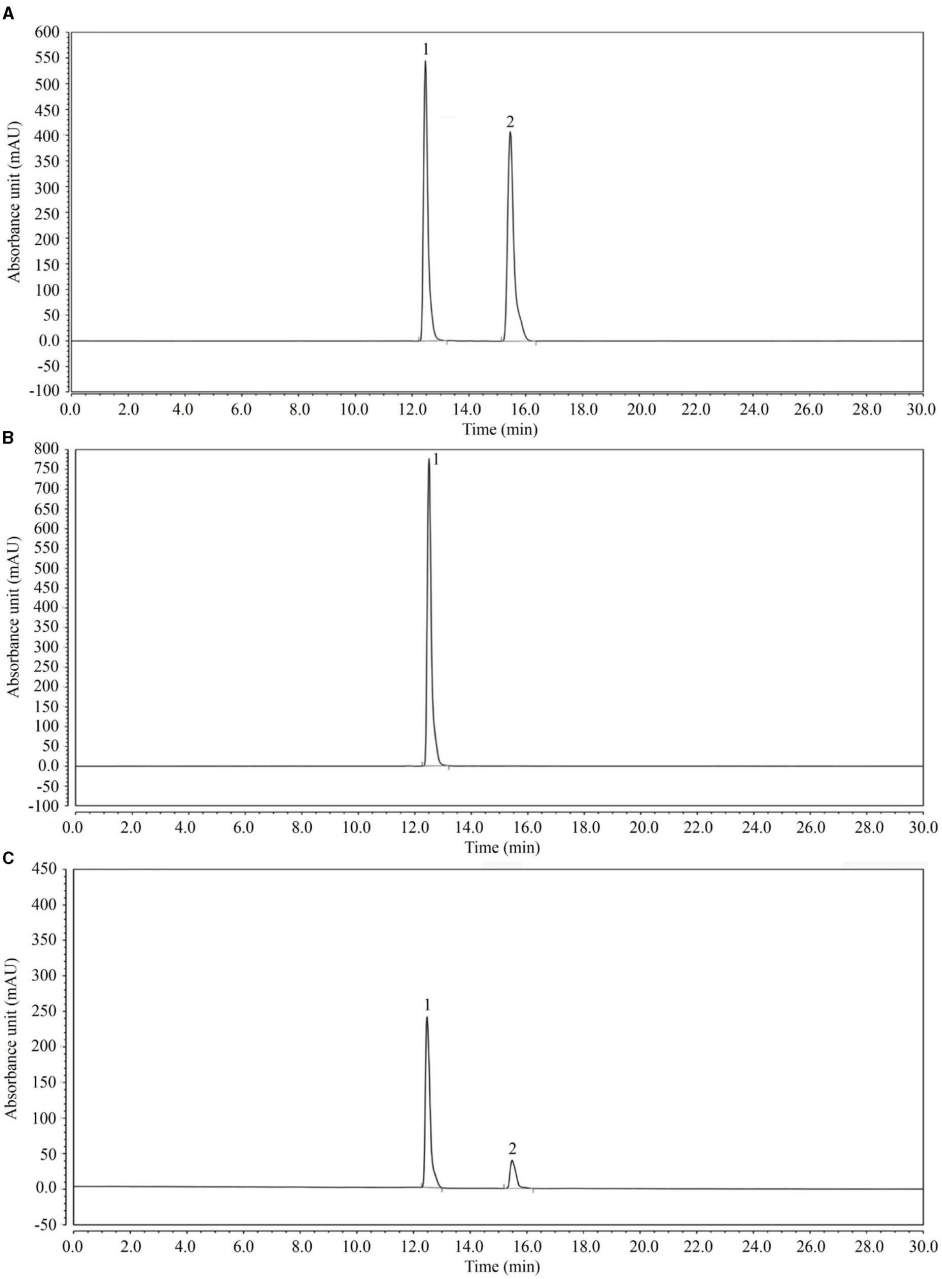
High-performance liquid chromatography (HPLC) analysis of the transformation experiment of geniposide to genipin by LcS. **(A)** Standards of geniposide and genipin; **(B)** geniposide and LcS reacted for 0 h; **(C)** geniposide and LcS reacted for 12 h. 1: geniposide, 2: genipin.

Through HPLC analysis (Figure 8), the results showed that after 12 h, LcS converted genipin glycoside into genipin, which was then able to efficaciously decrease inflammation and oxidation.

## Discussion

Sepsis is a deadly systemic disease. Sepsis and its complications often cause uncontrolled inflammation, tissue damage, and multiple organ failure, resulting in the death of patients in the ICU (Raman and Laupland, 2021). Similarly, in this study, sepsis also led to death of the mice, although LcS + geniposide increased sepsis survival rates, and LcS + geniposide enhanced dexamethasone efficacy. Sepsis can cause cognitive impairment that results in poor prognosis. Herein, we measured the cognitive ability of septic mice by an open field test (Cui et al., 2020). The Morris water maze test can also reflect the learning and cognitive ability of septic mice. In this study, the exploration and cognitive learning ability of the septic mice were affected through this experiment. However, LcS strengthened the efficacy of geniposide, which resulted in increased activity exploration and improved cognitive learning abilities of the septic mice.

Sepsis results in systemic inflammation, uncontrolled oxidative regulation, and neuronal apoptosis, leading to brain damage and cognitive impairment, which is considered to be a significant marker of sepsis and the mechanism of the disease (Yang B. et al., 2021). The hippocampus is an important regulator of learning, memory, emotion, and endocrine activities in the body (Zhao and Yu, 2021). This study also showed that sepsis induced the apoptosis of a large number of mouse nerve cells. We also found that LcS + geniposide protected the hippocampus and alleviated pathological changes. It is also suggested that oxidative stress and inflammatory reactions are important factors in neuronal apoptosis (Qiu et al., 2021; Su et al., 2021).

Tumor necrosis factor alpha, IL-6, and IL-1β are significant pro-inflammatory factors, which can lead to and promote the occurrence and development of sepsis (Li Q. et al., 2021). SOD and CAT are antioxidant enzymes that can inhibit oxidative stress injury, and MDA is a typical product of oxidative damage (Yang J. W. et al., 2021). An important method for alleviating sepsis is by reducing the imbalance and damage caused by oxidative stress and inflammatory reactions. By controlling the degree of oxidative stress and inflammation, nephrotoxicity can be alleviated, which will prevent systemic sepsis (Ma and Tang, 2021). In this study, we found that LcS promoted the conversion of geniposide into genipin, which reduced the TNF-α, IL-6, IL-1β, and MDA levels in the hippocampi of septic mice, and it increased the activities of SOD and CAT, indicating that the combination of geniposide and LcS decreased the inflammatory response and oxidative stress injury in mice with sepsis, with greater efficacy than geniposide utilized alone.

The activation of SIRT1 can alleviate normal cell apoptosis and reduce the degree of oxidation and inflammation, so as to reduce the systemic organ damage caused by sepsis (Liu J. Q. et al., 2021). SIRT1 has been shown to have beneficial effects on brain injury, ischemic injury, and other neurological diseases (Yao et al., 2021). This study showed that LcS + geniposide increased the expression of SIRT1, indicating that LcS + geniposide might ameliorate the cognitive dysfunction in septic mice *via* SIRT1 activation. SIRT1 activation exerts anti-apoptotic, anti-oxidant, and anti-inflammatory effects, and SIRT1 is closely related to the downstream deacetylation of FOXO1, p53, and NF-κB, which are mediated by SIRT1 (Tan et al., 2021). FOXO1 is regarded as a metabolic and antioxidant regulator, promoting SOD and CAT synthesis, with a particular effect on SOD1 and SOD2 that exist in animals (Li et al., 2019). SIRT1 directly deacetylates FoxO1, thus stimulating the gene expression of cell protection (Ren et al., 2020). p53 strongly expresses apoptosis, and is the first non-histone deacetylation target of SIRT1. p53 can regulate the function of deacetylation through SIRT1, thus inhibiting the apoptosis of normal cells (Liu et al., 2019). NF-κB participates in the development of sepsis by enhancing the transcription of proinflammatory cytokines, such as TNF-α, IL-6, and IL-1β. SIRT1 can inhibit NF-κB by deacetylating it, and thus plays a neuroprotective role (Li Y. et al., 2021). This study showed that LcS + geniposide decreased the expression of Ac-FOXO1, Ac-NF-κB, and Ac-p53 in mice with sepsis, confirming that LcS + geniposide affected the expression of Ac-FOXO1, Ac-NF-κB, and Ac-p53 by activating the expression of SIRT1, thereby regulating inflammation and oxidative stress, and even enabling the identification of cognitive dysfunction in mice with sepsis. The abilities of the mice in the Lc S + geniposide group were similar to those of the mice in the dexamethasone group.

**Figure 9:**
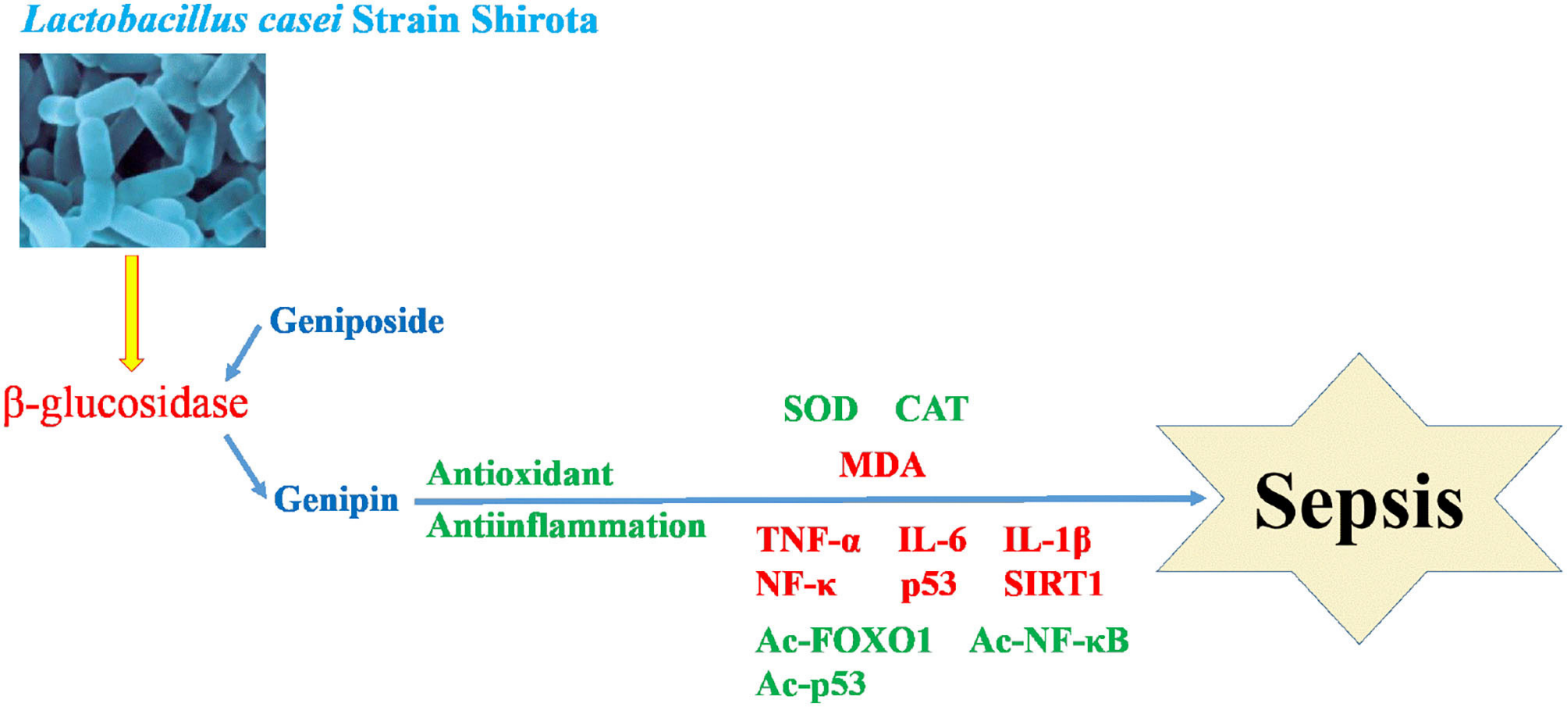
Mechanism of LcS promoting geniposide intervention in sepsis.

Mitochondria are the material center of cell synthesis and metabolism. Oxidative stress in sepsis can cause mitochondrial damage and dysfunction, and eventually lead to tissue and organ damage (Arulkumaran et al., 2018). Systemic inflammatory response syndrome (SIRS) refers to the systemic inflammatory response caused by any pathogenic factor acting on the body. Toxicosis is a systemic inflammatory reaction caused by excessive or uncontrolled inflammation (Pan et al., 2018). After sepsis occurs, lipopolysaccharides produced by bacteria inhibit the activity of antioxidant enzymes and produce excessive reactive oxygen species (ROS). The increase in ROS will lead to the destruction of the mitochondrial membrane lipid, and damage the activities of related enzymes and the structural integrity of the inner membrane (Crouser, 2004). Therefore, decreasing the mitochondrial oxidative stress response and tissue inflammation caused by oxidative stress is a strategy to be explored for sepsis (Zhang et al., 2020). In this study, we found that LcS can transform geniposide into genipin, which can promote anti-oxidation and anti-inflammation, and intervene in sepsis (Figure 9).

Genipin, as the intestinal metabolite of geniposide, is the main form of geniposide (Meng et al., 2013). Genipin has strong anti-inflammatory and anti-bacterial effects (Lim et al., 2008; Lelono et al., 2009). Because genipin is unstable, it is prone to change under natural conditions. Therefore, maintaining the stability of geniposide is important, so that LcS can transform geniposide into genipin, and then they can be used together when necessary, which is an efficient method for geniposide to exert its efficacy. This study also confirmed that LcS can transform geniposide into genipin and inhibit sepsis, and that the combination of geniposide and LcS is a feasible method to accomplish this.

## Conclusion

In summary, LcS + geniposide increased the rate of septic mouse survival, improved cognition in activity exploration and learning in mice with sepsis, reduced the rate of nerve cell apoptosis, and reduced inflammatory response and oxidative stress damage; the FOXO1, NF-κB, and p53 expressions were closely related to these responses. The experiments showed that LCS alone had no effect, the effect of geniposide when utilized alone was also weak, LcS greatly enhanced the geniposide effect on sepsis, and their effects were similar to the drug dexamethasone. The results of this study provide a new potential method for the treatment and regulation of sepsis, but the exact mechanism still requires further study.

## Data Availability Statement

The original contributions presented in the study are included in the article/supplementary material, further inquiries can be directed to the corresponding author/s.

## Ethics Statement

The animal study was reviewed and approved by The Ethical Committee for Animal Experiments at the Chongqing Medical University approved the study.

## Author Contributions

CM and LQ worked on animal experiments and co-authored this manuscript. YZ contributed to the data analysis. XT engaged in some data analysis. All authors checked the manuscript and agreed to the final version.

## Conflict of Interest

The authors declare that the research was conducted in the absence of any commercial or financial relationships that could be construed as a potential conflict of interest.

## Publisher's Note

All claims expressed in this article are solely those of the authors and do not necessarily represent those of their affiliated organizations, or those of the publisher, the editors and the reviewers. Any product that may be evaluated in this article, or claim that may be made by its manufacturer, is not guaranteed or endorsed by the publisher.

## Abbreviations

TNF-α: tumor necrosis factor alpha
IL-6: interleukin-6
IL-1β: interleukin-1 beta
ELISA: enzyme-linked immunosorbent assay
MDA: malondialdehyde
SOD: superoxide dismutase
CAT: catalase
NF-κB: nuclear factor kappa beta
IκB-α: inhibitor kappa B-alpha
SIRT1: sirtuin type 1
Ac-FOXO1: Ac-forkhead box O1
qPCR: quantitative polymerase chain reaction
HPLC: high-performance liquid chromatography
ICU: intensive care unit
LcS: *Lactobacillus casei* Strain Shirota
ICR: Institute of Cancer Research
FITC: fluorescein isothiocyanate isomer
PCR: polymerase chain reaction
GAPDH: glyceraldehyde-3-phosphate dehydrogenase
RIPA: radio immunoprecipitation assay
BCA: bicinchoninic acid.
